# Reduced CD40 expression in B cell subsets of individuals with radiologically isolated syndrome

**DOI:** 10.3389/fimmu.2025.1680233

**Published:** 2025-12-01

**Authors:** Christian W. Keller, Kerstin Stein, Andreu Vilaseca, Luisa M. Villar, Gary Álvarez-Bravo, Maria Protopapa, Josef Shin, Nicolás Fissolo, José Enrique Martínez-Rodríguez, Ana Quiroga-Varela, Alexis García-Sarreón, Lucía Gutiérrez, Luciana Midaglia, Xavier Montalban, Jan D. Lünemann, Manuel Comabella

**Affiliations:** 1 Department of Neurology, University Hospital Münster, Münster, Germany; 2 Department of Neurology and Neurophysiology, University Medical Center Freiburg, Freiburg, Germany; 3 Servei de Neurologia and Centre d’Esclerosi Múltiple de Catalunya (Cemcat), Institut de Recerca Vall d’Hebron (VHIR), Hospital Universitari Vall d’Hebron, Universitat Autònoma de Barcelona, Barcelona, Spain; 4 Departments of Neurology and Immunology, Hospital Universitario Ramón y Cajal, Instituto Ramón y Cajal de Investigación Sanitaria, Madrid, Spain; 5 Unit of Neuroimmunology and Multiple Sclerosis of Girona (UNIEMTG), Hospital Universitari de Girona Dr. Josep Trueta & Hospital Santa Caterina, Neurodegeneration & Neuroinflammation group, Institut d’Investigació Biomèdica de Girona (IDIBGI-CERCA), Salt, Spain; 6 Red de Enfermedades Inflamatorias, Carlos III Health Institute, Madrid, Spain; 7 Department of Medical Sciences, Faculty of Medicine, University of Girona, Girona, Spain; 8 Department of Neurology, Research Center for Immunotherapy (FZI) and Focus Program Translational Neuroscience (FTN), Rhine Main Neuroscience Network (Rmn2), University Medical Center of the Johannes Gutenberg University Mainz, Mainz, Germany; 9 Center for Networked Biomedical Research on Neurodegenerative Diseases (CIBERNED) - ISCIII, Madrid, Spain; 10 Neurology Department, Hospital del Mar Medical Research Institute (IMIM), Barcelona, Spain

**Keywords:** multiple sclerosis, B cells, radiologically isolated syndrome, CD40, biomarkers

## Abstract

**Introduction:**

B cells play a central role in multiple sclerosis (MS) pathogenesis, yet their activation profiles during the earliest disease stages remain incompletely characterized. We aimed to investigate the expression of the activation markers CD40 and CD71 across peripheral B cell subsets in individuals with radiologically isolated syndrome (RIS), and compare them to those with clinically isolated syndrome or MS (CIS/MS) and healthy controls (HC).

**Methods:**

Peripheral blood cells from individuals with RIS (n=21), CIS/MS (n=15), and HC (n=15) were analyzed by flow cytometry. CD40 and CD71 expression was assessed across major B cell subsets. Epstein-Barr virus (EBV) serologies were measured to explore associations with activation marker expression.

**Results:**

CD40 expression was reduced on transitional, naïve, B cell subsets in RIS compared to CIS/MS and HC (p<0.001). CD71 expression remained stable across groups. CD40 expression was preserved in plasmablasts and regulatory B cells. No significant associations were observed between CD40 levels and EBV antibody titers.

**Discussion:**

In this cross-sectional cohort, CD40 expression was lower on transitional, naïve, and memory B cell subsets in RIS compared to CIS/MS and HC, whereas plasmablasts and regulatory B cells were comparable. This pattern is consistent with an altered costimulatory state in early, asymptomatic disease and warrants longitudinal studies to determine its clinical relevance.

## 1 Introduction

Multiple sclerosis (MS) is a chronic inflammatory and neurodegenerative disorder of the central nervous system (CNS) characterized by focal demyelination, axonal loss, and progressive neurological dysfunction. Although traditionally regarded as a T cell-mediated disease, a large body of experimental and clinical evidence has established a pivotal role for B cells in MS pathogenesis (1–3). B cells exert diverse functions, including antigen presentation, cytokine secretion, antibody production, and the regulation of T cell responses. The profound efficacy of B cell-depleting therapies in relapsing and progressive MS has transformed disease management and firmly established these cells as central drivers of pathology rather than passive bystanders (4).

Peripheral B cells comprise heterogeneous subsets with distinct developmental and functional profiles in MS. Furthermore, the disease features compartmentalized B cell immunity in the meninges and perivascular spaces that associates with cortical demyelination and worse clinical course, implicating B cells not merely as antibody producers but as antigen-presenting and cytokine-secreting orchestrators of CNS autoimmunity (2). Histopathology demonstrates ectopic B cell-rich infiltrates and tertiary lymphoid-like structures in progressive MS, with meningeal inflammation tracking with subpial cortical pathology and disability acceleration (5, 6). CSF and lesion repertoire studies further show antigen-experienced, clonally expanded B cells bidirectionally exchanged across the blood–brain barrier, supporting sustained intrathecal B cell activation (7–9).

Although intrathecal and meningeal B cell populations appear to be key drivers of CNS-compartmentalized inflammation, they are not readily accessible for routine analysis. Peripheral blood therefore provides a practical and informative window into systemic B cell biology across disease stages. Within the circulation, distinct subsets can be delineated that mirror developmental and functional heterogeneity observed in the CNS. Transitional B cells are enriched in IL-10–competent cells with regulatory potential, whereas naïve B cells provide a precursor reservoir that can supply autoreactive clones under permissive cues. Memory B cells efficiently present antigen, produce pro-inflammatory cytokines (such as TNF, IL-6, GM-CSF), and populate CNS niches; their expansion correlates with inflammatory activity and is impacted by anti-CD20 therapies. Plasmablasts/plasma cells contribute to intrathecal oligoclonal IgG and may sustain chronic CNS inflammation independent of peripheral B cell depletion (1, 2, 10). Collectively, these data support a model of subset-specific imbalance, with pro-inflammatory memory/plasmablast compartments predominating and regulatory compartments being quantitatively or functionally constrained (1, 10).

CD40 and CD71 provide complementary windows into these processes. CD40, a TNFR-superfamily costimulatory receptor on B cells, integrates CD4^+^ T cell help to drive germinal center formation, isotype switching, and pro-inflammatory cytokine production (11, 12). The receptor also governs critical B cell fate decisions including survival, proliferation, and memory B cell formation through interaction with CD154 on T cells. While CD40 stimulation can promote memory B cell generation, evidence also shows it may block plasma cell differentiation, indicating that the precise role of CD40 signaling in directing B cell differentiation remains context-dependent and not yet fully resolved (13).

In MS, dysregulated B cell costimulation and heightened T-B collaboration are consistently implicated by human tissue, CSF, and interventional data; anti-CD20 therapies attenuate T cell proliferation and pro-inflammatory myeloid responses in part by depleting antigen-presenting and cytokine-producing memory B cells (1, 10). Measuring CD40 on discrete B cell compartments thus gauges costimulatory set-points that may shift before clinical onset. In contrast, CD71 (transferrin receptor-1) is a canonical marker of lymphocyte proliferation and metabolic activation; its induction accompanies cell-cycle entry and effector differentiation (14–16). Parallel assessment of CD71 alongside CD40 therefore may help distinguish generalized activation and metabolic readiness from targeted costimulatory modulation at the subset level.

Positioning these readouts along the RIS - CIS - MS continuum is informative. RIS, a pre-symptomatic radiological stage with measurable conversion risk, offers a window on early immune deviations potentially preceding clinical disease. Subset-resolved analysis of CD40 and CD71 across these stages can reveal whether alterations in expression arise preclinically in peripheral blood and whether they are maintained, normalized, or compartmentalized with clinical onset.

## 2 Materials and methods

### 2.1 Subjects

The study included 15 patients whose blood was collected at the time of the first demyelinating event or clinically isolated syndrome (CIS). Of these, 10 patients fulfilled the 2017 McDonald criteria for relapsing-remitting MS (17), whereas 5 remained a CIS at the time of the last follow-up. The study also included 21 individuals with radiologically isolated syndrome who fulfilled the 2017 McDonald criteria for dissemination in space at the diagnostic magnetic resonance imaging (18). Of these, 8 developed symptoms during follow-up and were diagnosed with relapsing-remitting MS (RIS_MS), while 13 remained asymptomatic (RIS). None of the patients or individuals with RIS received immunomodulatory or immunosuppressive therapies prior to inclusion in the study. A group of 15 healthy controls (HC) was also included for comparison. The study was approved by the hospital ethics committee, and all participants provided written informed consent. The main demographic and clinical characteristics of the patients and HC are summarized in **Supplementary Table S1**.

### 2.2 B cell immunophenotyping

Surface marker expression on B cell subsets was quantified by flow cytometry in frozen peripheral blood mononuclear cells (PBMC) isolated from patients and controls using Ficoll-Isopaque density gradient centrifugation (Gibco BRL, Life Technologies LTD, UK) and stored in liquid nitrogen until use. Briefly, dead cells were excluded using Fixable Aqua Dead Cell Stain Kit (BioLegend, San Diego, CA). Frozen patient and control PBMC were quickly thawed, washed and immediately transferred into FACS tubes. Prior to fluorescent antibody staining, Fc receptor blocking reagent (Human TruStain FcX™ Fc Receptor Blocking Solution [BioLegend]) was added to all samples for 10 min on ice to prevent unspecific Fc-mediated binding of Abs to FcRs. Cells were suspended in phosphate-buffered saline 0.01% sodium azide containing the respective fluorochrome-labeled antibodies for detecting surface molecules and incubated for 30 min on ice in the dark. Cells were then washed and suspended in phosphate-buffered saline 0.01% sodium azide and acquired using CytExpert 2.3 software on a CytoFLEX flow cytometer (Beckman Coulter, Brea, CA). All analyses were performed with FlowJo 10 (Tree Star Inc., Ashland, OR). A summary of the antibodies used for immunophenotyping is provided in **Supplementary Table S2**.

To identify B cell subpopulations, we employed a gating strategy that delineates all major B cell subsets based on surface marker expression (19–27) (**Supplementary Figures S1, S2**). Median Fluorescence Intensities (MFI) of CD40 and CD71 were determined for the following B cell subsets analyzed in the study:

Transitional B cells: CD19+CD38hiCD24+IgM+IgD+CD27-CD1d-. Naïve B cells: CD19+CD38+IgD+CD27-. Unswitched memory B cells: CD19+CD38+CD27+IgD+. Switched memory B cells: CD19+CD38+CD27+IgD-. Plasmablasts: CD19+CD38hiCD27+CD24-CD21-. Regulatory B cells (Bregs): CD19+CD38+CD1d+CD24+CD27+.

### 2.3 Epstein-barr virus serologies

Viral IgG antibodies against EBV-encoded nuclear antigen-1 (EBNA1) and the viral capsid antigen (VCA) were determined by commercially available ELISAs in serum samples obtained concurrently with the PBMC, as previously described (28, 29).

### 2.4 Statistical analysis

Statistical analyses were performed using RStudio (Posit Software, PBC) running R version 4.4.2. Group differences in CD40 and CD71 expression across B cell subsets among HC, patients with CIS/MS and individuals with RIS were evaluated using the Kruskal-Wallis test. When the global test was significant (p < 0.05), pairwise comparisons were performed using Dunn’s test. Comparisons of activation marker expression between CIS and MS patients, as well as between RIS and RIS_MS patients, were conducted using the Mann-Whitney U test. Associations between activation marker expression and EBV humoral immune responses were assessed using Spearman’s rank correlation. Graphs were generated using the ggplot2 package.

## 3 Results

No significant differences were observed in age or sex distribution between groups. The mean (standard deviation) follow-up time was 6.1 (2.5) for patients with CIS/MS and 4.8 (3.2) for individuals with RIS, respectively (**Supplementary Table S1**).

**Figure 1:**
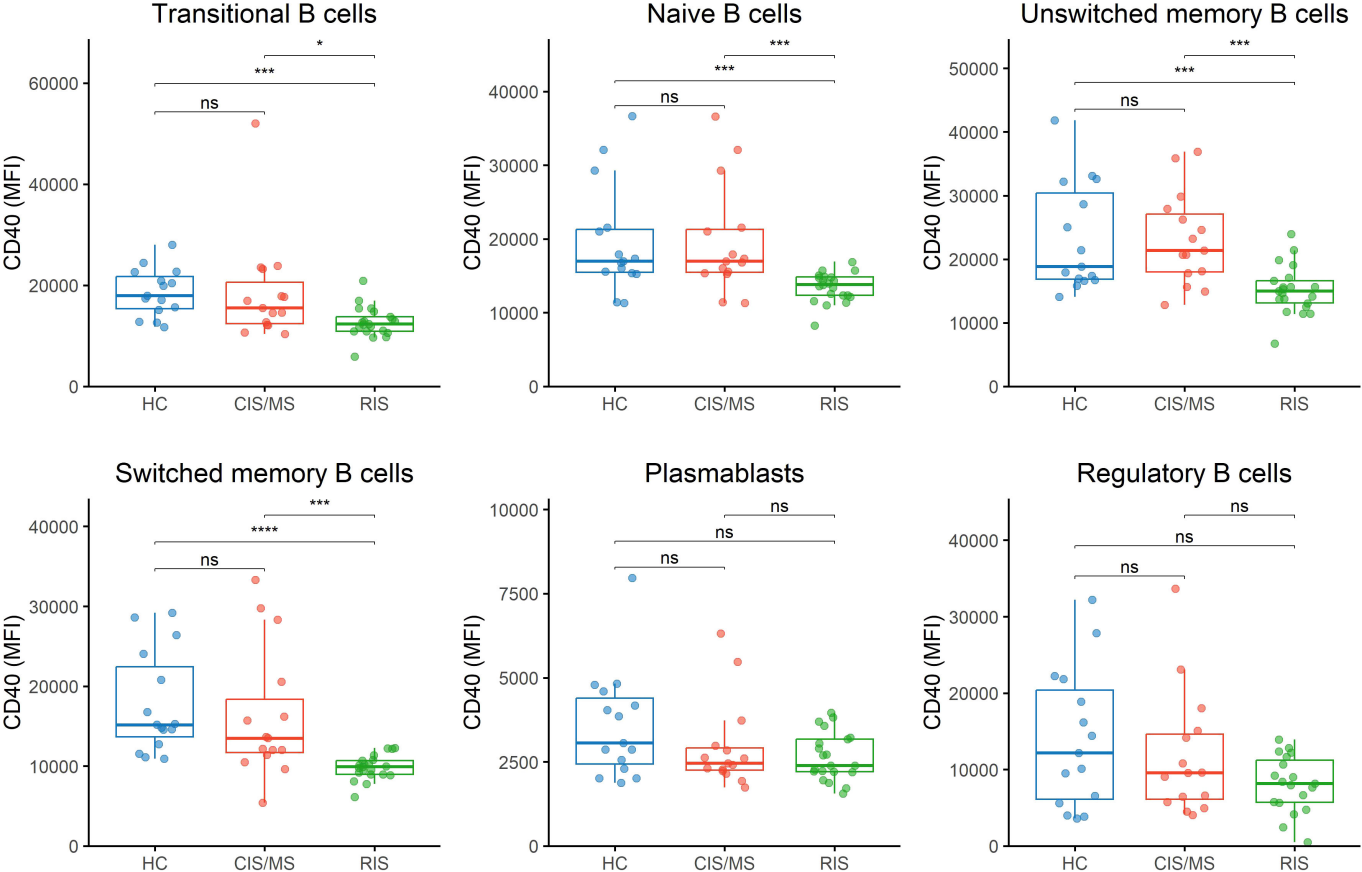
CD40 expression across B cell subsets. CD40 expression on B cell subsets was determined by flow cytometry in peripheral blood mononuclear cells from CIS/MS patients (n=15), individuals with RIS (n=21), and HC (n=15). Transitional B cells: CD19^+^CD38^hi^CD24^+^IgM^+^IgD^+^CD27^−^CD1d^−^. Naïve B cells: CD19^+^CD38^+^IgD^+^CD27^−^. Unswitched memory B cells: CD19^+^CD38^+^CD27^+^IgD^+^. Switched memory B cells: CD19^+^CD38+CD27^+^IgD^−^. Plasmablasts: CD19^+^CD38^hi^CD27^+^CD24^−^CD21^−^. Regulatory B cells: CD19^+^CD38^+^CD1d^+^CD24^+^CD27^+^. CIS/MS: refers to the entire group of patients with clinically isolated syndrome (CIS) and MS. RIS, refers to the entire group of individuals with radiologically isolated syndrome. HC, healthy controls. MFI, median fluorescence intensity. *p<0.05. ***p<0.001. ****p<0.0001. ns, non-significant.

**Figure 2:**
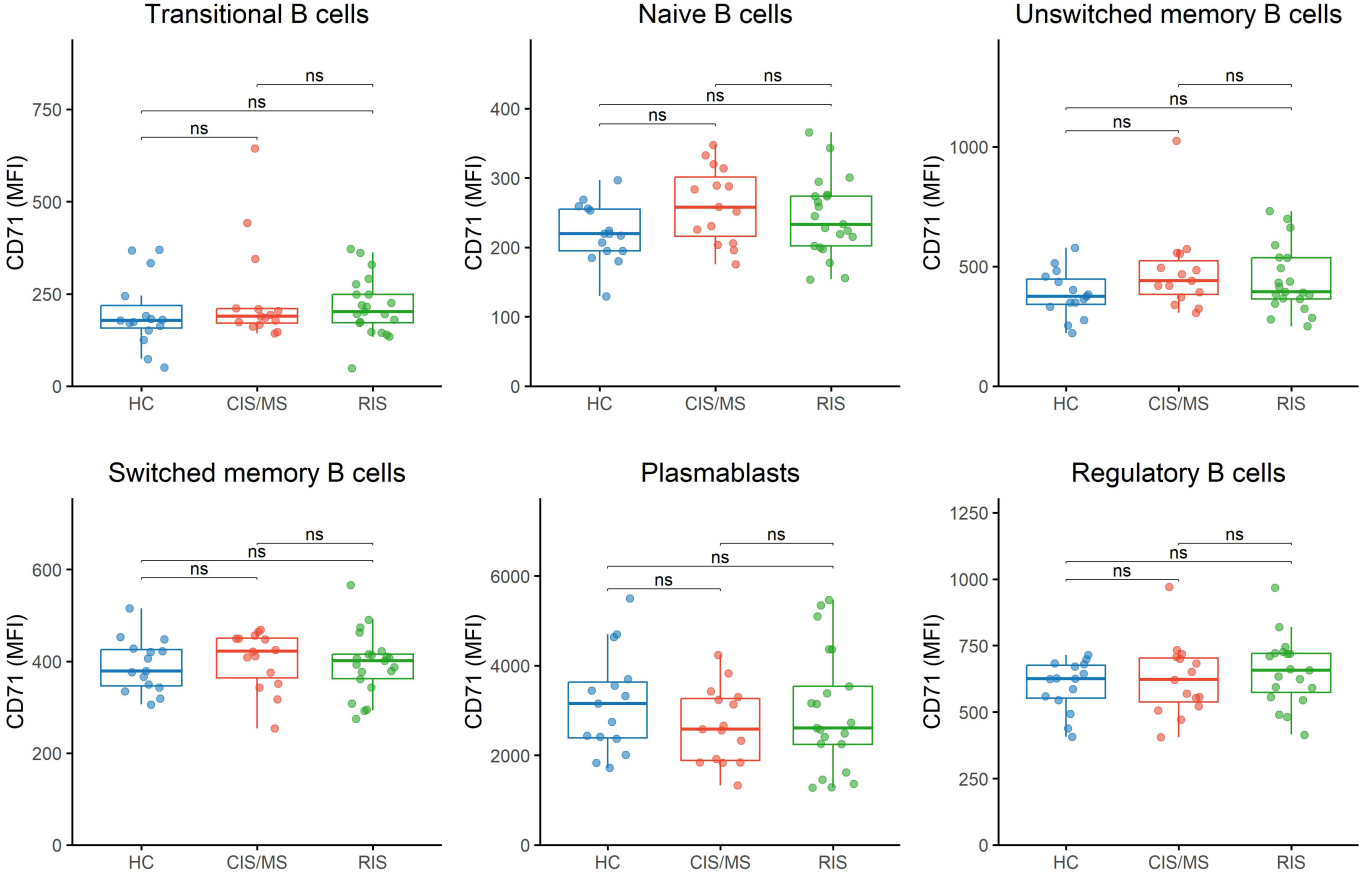
CD71 expression across B cell subsets. CD71 expression on B cell subsets was determined by flow cytometry in peripheral blood mononuclear cells from CIS/MS patients (n=15), individuals with RIS (n=21), and HC (n=15). B cell subsets were defined as in **Figure 1**. CIS/MS: refers to the entire group of patients with clinically isolated syndrome (CIS) and MS. RIS, refers to the entire group of individuals with radiologically isolated syndrome. HC, healthy controls. MFI, median fluorescence intensity. ns, non-significant.

As shown in **Figure 1**, CD40 expression was decreased in transitional B cells, naïve B cells, and both unswitched and switched memory B cells in individuals with RIS compared to the CIS/MS group and HC (p<0.001 for all comparisons, except for the comparison between the RIS and CIS/MS groups, which showed p<0.05), whereas no significant differences were observed for plasmablasts or Bregs. In contrast, CD71 expression was similarly distributed across the different groups (**Figure 2**; **Supplementary Figure S3**).

**Figure 3:**
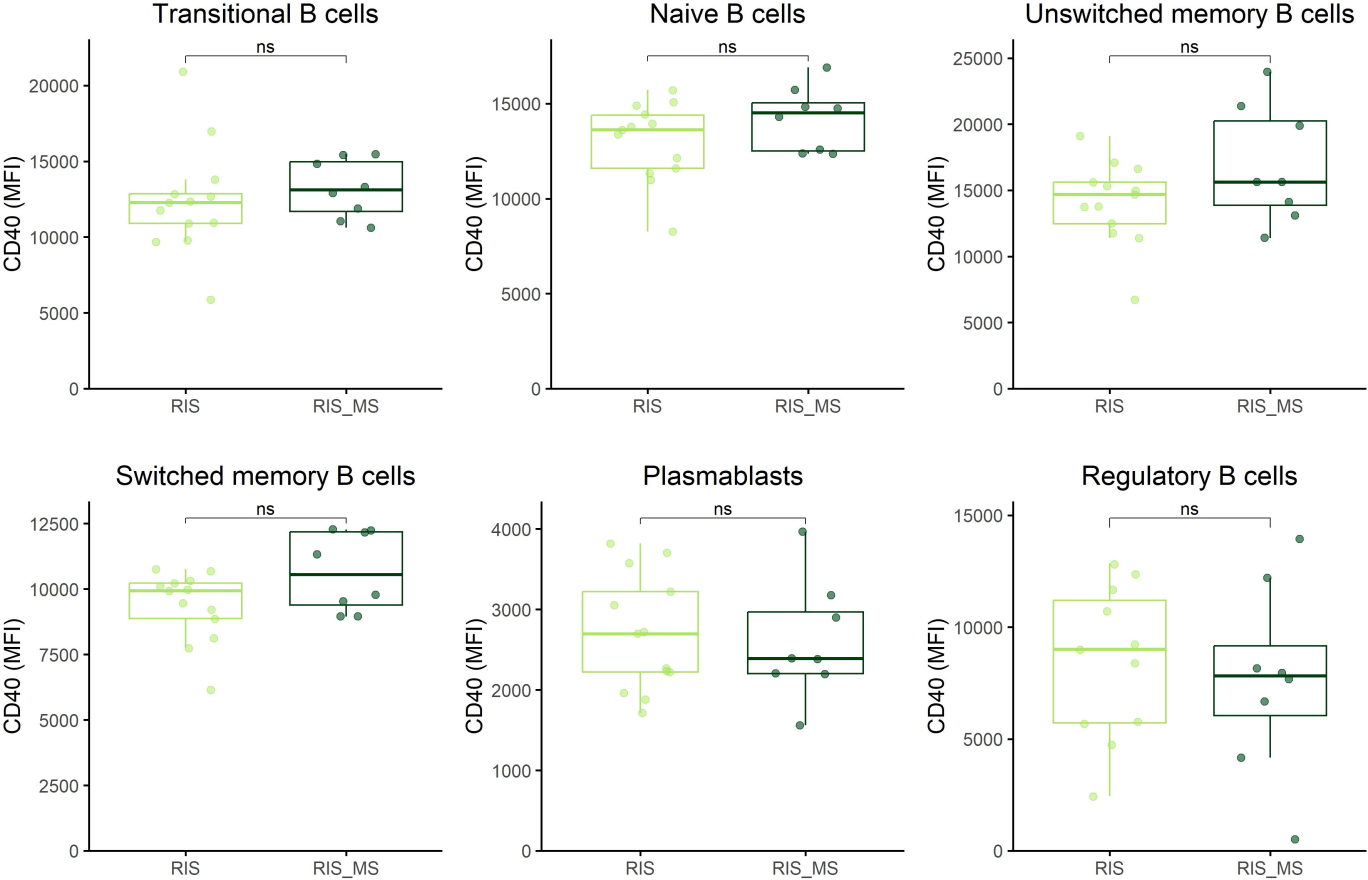
CD40 expression across B cell subsets in individuals with RIS. CD40 expression on B cell subsets was determined by flow cytometry in peripheral blood mononuclear cells from individuals with RIS who developed MS symptoms (RIS_MS; n=8) and those who did not (RIS; n=13). B cell subsets were defined as in **Figure 1**. RIS, radiologically isolated syndrome; MFI, median fluorescence intensity; ns, non-significant.

**Figure 4:**
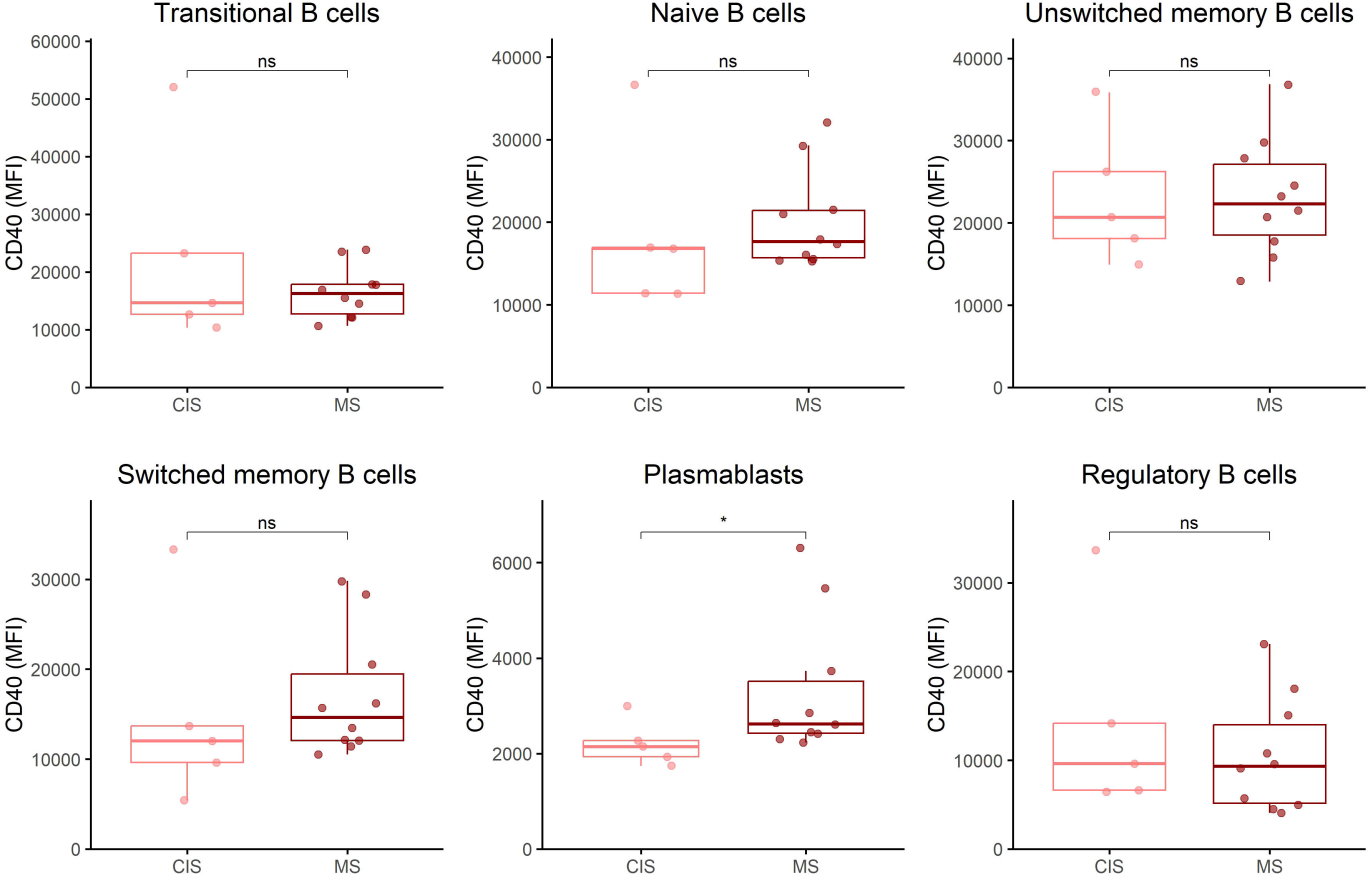
CD40 expression across B cell subsets in individuals with CIS and MS. CD40 expression on B cell subsets was determined by flow cytometry in peripheral blood mononuclear cells from patients with CIS (n=5) and patients with MS (n=10). B cell subsets were defined as in **Figure 1**. CIS, clinically isolated syndrome; MFI, median fluorescence intensity. *p<0.05. ns, non-significant.

When the RIS group was divided into individuals who developed MS symptoms during follow-up and those who did not, no significant differences were observed between the groups in CD40 and CD71 expression across the different B cell subsets (**Figure 3**; **Supplementary Figure S4**, respectively). When the CIS/MS group was split into patients with CIS and patients with MS, a significant increase in CD40 expression was observed only in plasmablasts of MS patients compared to CIS patients (p=0.04), whereas no differences were found for the other B cell subsets (**Figure 4**) or for CD71 expression (**Supplementary Figure S5**).

When comparing the distribution of circulating B cell subsets across HC, CIS, and RIS (**Supplementary Figure S6**), we found the frequency of naïve B cells to be significantly increased in the RIS group compared with HC (p=0.02). By contrast, unswitched and switched memory, as well as transitional B cell subsets, showed no significant differences between groups. Bregs were reduced in the RIS group relative to HC (p=0.02) and the CIS/MS group (p=0.01), whereas plasmablast frequencies did not differ significantly across groups. These findings suggest that RIS is associated with a skewing of the peripheral B cell compartment toward naïve phenotypes and reduced Breg-enriched populations, without changes in transitional, unswitched memory, switched memory, or plasmablast subsets.

**Figure 5:**
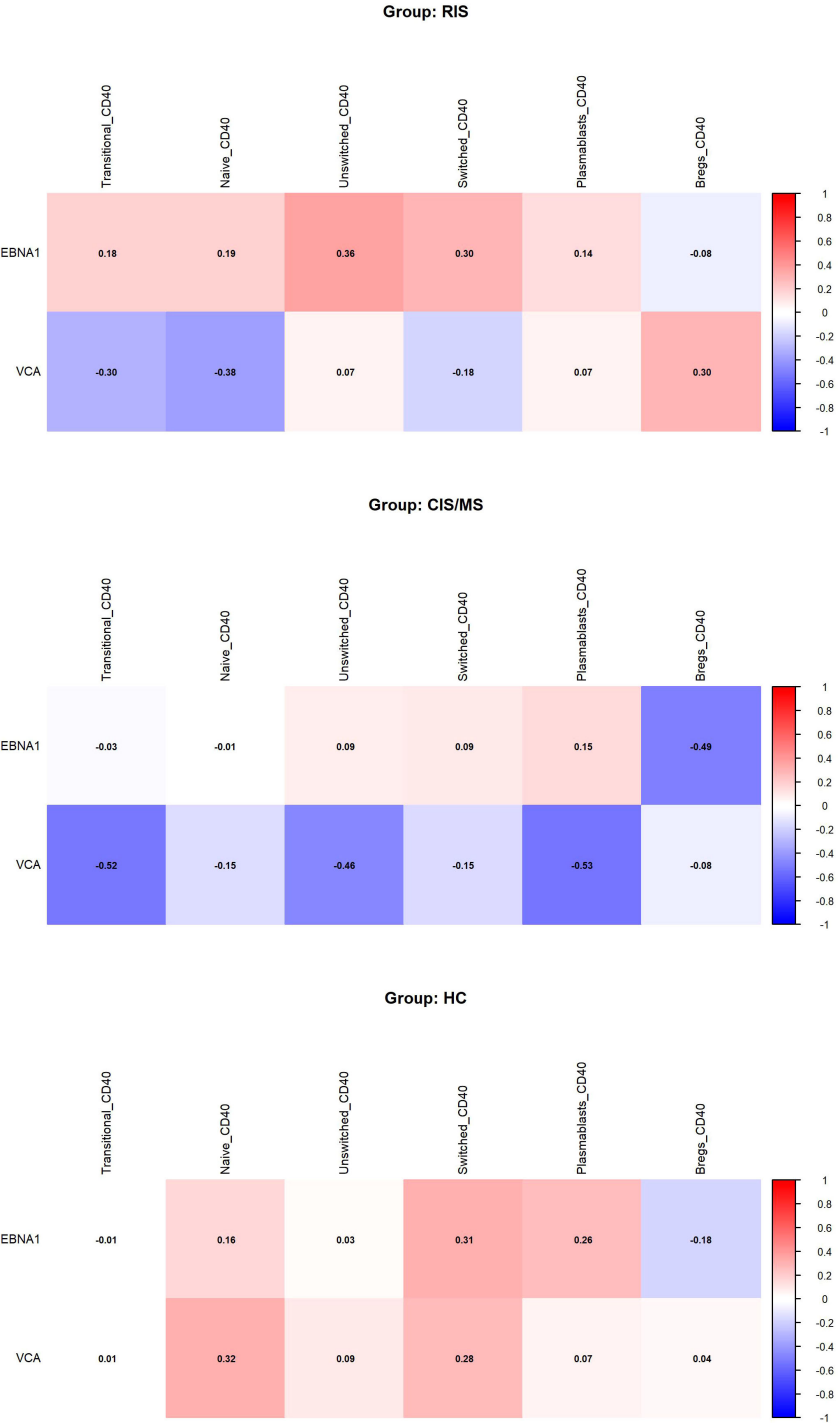
Correlations between humoral immune responses to EBV and CD40 expression in B cell subsets. Heatmap showing Spearman correlation coefficients between antibody levels against EBNA1 and VCA and CD40 expression in B cell subsets. Positive correlations are represented in shades of red, and negative correlations in shades of blue, according to the color scale displayed on the right side of the figure. None of the correlations were statistically significant. EBNA1, Epstein-Barr virus-encoded nuclear antigen-1; VCA, viral capsid antigen; CIS/MS, refers to the entire group of patients with clinically isolated syndrome (CIS) and MS; RIS, refers to the entire group of individuals with radiologically isolated syndrome; HC, healthy controls.

Considering the role of EBV in MS and the fact that B cells are the main reservoir for the virus (30, 31), we also investigated the potential association between humoral immune responses against EBV and CD40 expression in B cell subsets. As shown in **Figure 5**, no statistically significant correlations were observed between antibody levels against EBNA1 or VCA and CD40 expression in B cell subsets from patients with CIS/MS, individuals with RIS, or HC.

## 4 Discussion

Our study identifies distinct patterns of B cell activation in individuals with RIS, an early preclinical stage of MS, characterized by incidental MRI findings suggestive of demyelination in the absence of clinical symptoms (32). Specifically, we observed a significant downregulation of the costimulatory molecule CD40 across transitional, naïve, and memory B cell subsets in RIS, while the expression of CD71, a marker of metabolic activation and proliferation, remained stable across all groups. These findings provide novel insights into the immunoregulatory dynamics of B cells during the earliest detectable phases of MS pathogenesis.

CD40 plays a central role in B cell-T cell interactions by promoting B cell activation, germinal center formation, immunoglobulin class switching, and pro-inflammatory cytokine production through engagement with CD40L on activated CD4^+^ T cells (11). In the context of autoimmunity, upregulated CD40 signaling has been implicated in sustaining aberrant immune responses and driving chronic inflammation (33, 34). Therefore, the observed reduction in CD40 expression across multiple B cell subsets in RIS may reflect an early, adaptive counter-regulatory mechanism aimed at limiting excessive immune activation during the asymptomatic phase of disease.

Interestingly, this downregulation was not observed in plasmablasts or Bregs, which retained CD40 expression. This suggests a differentiation-state-dependent regulation of CD40, potentially linked to the distinct functional trajectories of these subsets. Plasmablasts, being short-lived antibody-producing cells, may require sustained CD40 expression to complete their differentiation, while Bregs may utilize CD40 to maintain tolerogenic signaling pathways. These findings align with prior work showing that B cell subsets exhibit highly context-dependent expression of activation markers depending on their maturation state and microenvironmental cues (35).

The preservation of CD71 expression across all B cell populations, including in RIS, indicates that the metabolic and proliferative potential of these cells remains intact during early MS. CD71 facilitates iron uptake, which is critical for mitochondrial function and DNA synthesis during B cell proliferation (14). Its stable expression may imply ongoing immune surveillance or basal B cell activation, rather than a pronounced inflammatory response. These results are consistent with studies in other immune settings showing that CD71 is upregulated upon activation but not necessarily modulated by immunoregulatory cues in the same manner as costimulatory molecules like CD40 (16).

Unexpectedly, RIS patients exhibited lower CD40 expression than healthy donors across several B cell compartments. This finding suggests that downregulation of CD40 may occur early, before clinical onset, potentially as a genetic or adaptive response to nascent immune dysregulation. By contrast, this effect was absent in CIS/MS, raising the possibility that CD40 modulation is stage-dependent and overshadowed by subsequent inflammatory processes once clinical disease emerges.

Importantly, our data do not support a direct relationship between CD40 downregulation and EBV serostatus, as no significant correlations were observed. This suggests that the observed CD40 modulation is not merely a reflection of anti-viral B cell responses. Given the extensive literature linking EBV to MS pathogenesis, including its role in shaping B cell phenotypes and contributing to the latent reservoir of autoreactive clones (31), further studies are warranted to explore whether EBV reactivation or epitope spreading contributes to phenotypic shifts in B cells during the RIS phase.

The immunobiological significance of CD40 downregulation in RIS remains speculative but may represent an attempt by the immune system to establish peripheral tolerance or avoid excessive antigen-driven expansion during a state of chronic, low-level CNS immune activation. Previous studies have shown that B cell abnormalities can be detected early in MS patients sometimes even prior to the first clinical episode, with memory B cells demonstrating increased antigen presentation capacity and altered cytokine production profiles (36–38). In this context, our findings raise the possibility that early downregulation of CD40 may constitute a transient compensatory mechanism that is ultimately lost during progression to CIS or relapsing-remitting MS, allowing for the unchecked formation of autoreactive B cell–T cell interactions and germinal center activity in peripheral lymphoid tissues or CNS-associated meningeal structures (39).

Moreover, it remains unclear what immunological or environmental signals drive CD40 modulation in early MS. Candidate mechanisms include cytokine milieu alterations (e.g., elevated IL-10 or TGF-β), changes in microbiome composition, or antigen-independent BCR signaling. Animal models of MS and human studies suggest that CD40 expression is tightly regulated by both intrinsic transcriptional programs and extrinsic stimuli, including contact with follicular helper T cells and antigen-presenting cells (33, 40, 41). Therefore, understanding the upstream regulatory networks influencing CD40 expression may offer new opportunities for therapeutic modulation, especially in the preclinical or prodromal phases of MS.

Finally, our findings may have clinical implications. The observation of reduced CD40 expression in RIS suggests that altered costimulatory signaling could accompany early, asymptomatic stages of MS and may be relevant to the trajectory of disease evolution.

Conversely, the loss of this phenotype may signal impending conversion to clinical MS. However, a limitation of our study is the small sample size, highlighting the need for studies including a larger number of patients. In addition, longitudinal studies incorporating serial immunophenotyping, neuroimaging, and neurofilament light chain and GFAP measurements will be useful to determine the prognostic value of CD40 expression dynamics.

In conclusion, our study highlights a previously underappreciated modulation of B cell activation in the earliest detectable phase of MS. The selective downregulation of CD40 across multiple B cell subsets in RIS may represent an endogenous attempt to suppress pathogenic immune activity before the onset of clinical symptoms. Elucidating the molecular and environmental drivers of this phenotype may not only deepen our understanding of MS pathogenesis but also open new avenues for early intervention and biomarker development.

## Data Availability

The raw data supporting the conclusions of this article will be made available by the authors, without undue reservation.
